# Methyl 3,4-Dihydroxybenzoate Enhances Resistance to Oxidative Stressors and Lifespan in *C. elegans* Partially via *daf-2/daf-16*

**DOI:** 10.3390/ijms19061670

**Published:** 2018-06-05

**Authors:** Xiang-Nan Mi, Li-Fang Wang, Yang Hu, Jun-Ping Pan, Yi-Rong Xin, Jia-Hui Wang, Hai-Ju Geng, Song-Hui Hu, Qin Gao, Huan-Min Luo

**Affiliations:** 1 Department of Pharmacology, School of Medicine, Jinan University, Guangzhou 510632, China; mrsmanjusaka@163.com (X.-N.M.); wlifang001@163.com (L.-F.W.); yanghu0325@163.com (Y.H.); panjunpingjnu319@163.com (J.-P.P.); xinyirong@yeah.net (Y.-R.X.); wjh8623@126.com (J.-H.W.); jiapin@outlook.com (H.-J.G.); jinanyaolihu326@163.com (S.-H.H.); tgaoq@jnu.edu.cn (Q.G.); 2 School of Nursing, Guangdong Pharmaceutical University, Guangzhou 510632, China; 3 Institute of Brain Sciences, Jinan University, Guangzhou 510632, China

**Keywords:** Methyl 3,4-dihydroxybenzoate, ageing, *Caenorhabditis elegans*, *daf-2*, *daf-16*/*FoxO*, oxidative stress

## Abstract

Genetic studies have elucidated mechanisms that regulate aging; however, there has been little progress in identifying drugs that retard ageing. *Caenorhabditis elegans* is among the classical model organisms in ageing research. Methyl 3,4-dihydroxybenzoate (MDHB) can prolong the life-span of *C. elegans*, but the underlying molecular mechanisms are not yet fully understood. Here, we report that MDHB prolongs the life-span of *C. elegans* and delays age-associated declines of physiological processes. Besides, MDHB can lengthen the life-span of *eat-2* (ad1113) mutations, revealing that MDHB does not work via caloric restriction (CR). Surprisingly, the life-span–extending activity of MDHB is completely abolished in *daf-2* (e1370) mutations, which suggests that *daf-2* is crucial for a MDHB-induced pro-longevity effect in *C. elegans*. Moreover, MDHB enhances the nuclear localization of *daf-16*/*FoxO*, and then modulates the expressions of genes that positively correlate with defenses against stress and longevity in *C. elegans*. Therefore, our results indicate that MDHB at least partially acts as a modulator of the *daf-2/daf-16* pathway to extend the lifespan of *C. elegans*, and MDHB might be a promising therapeutic agent for age-related diseases.

## 1. Introduction

“Every man desires to live long but no man wishes to be old,” Jonathan Swift said in the 17th century. Although treatments for aging would be desirable, the development of such treatments is challenging [1,2]. Approaches based on random screens of potential treatments require relevant and practicable assays of aging, but the time and effort needed to measure aging are substantial obstacles. To address these challenges, we exploited the *C. elegans* model system.

*Caenorhabditis elegans* (*C. elegans*) has been exploited as a classical model organism in aging research for its simple biology, self-fertilization, short lifespan, ease of culture, and ease of genetic analysis from the early 1970s onward [3,4,5,6]. The observation that *C. elegans* has only 35% homology to human genes has long been seen as one of its chief deficits in anti-aging research [7,8]. Surprisingly, it has not prevented nematodes from becoming a classical model organism in biogerontological studies [8].

Methyl 3,4-dihydroxybenzoate (MDHB) has been reported to have antioxidant, neurotrophic, and neuroprotective effects [9,10,11,12]. We previously reported that MDHB might extend the adult lifespan of *C. elegans* partly through the *W06A7.4* gene [13]. However, the exact mechanisms of the lifespan-extending activity of MDHB in *C. elegans* remain unclear. The studies described here were conducted to investigate the potential mechanisms of MDHB in lifespan extension.

Several genetic and environmental manipulations are associated with the life-span of *C. elegans* [14], such as the insulin/IGF-1 signaling (IIS) pathway, known as the most typical pathway regulating lifespan in many creatures. Confined IIS signaling regulates the insulin/IGF-1 transmembrane receptor (IGFR) ortholog *daf-2*; the core transcriptional factor forkhead box O (*FoxO*) transcription factor *daf-16* and its interaction with additional transcriptional factors in the nucleus could prolong the lifespan of nematodes and promote health in mammals [15,16,17,18]. In addition, the oxidative stress pathway that represents an imbalance between toxic reactive oxygen species (ROS) and antioxidant systems is considered to be connected to lifespan regulation in worms [19]. Furthermore, dietary restriction (DR) was proved to be the only intervention that successfully prolongs lifespan in mammals [20]. To investigate the relationships between MDHB and these regulators of aging, we exploited the *C. elegans* model system to investigate the effects and potential mechanisms of MDHB on the longevity of *C. elegans*.

The results suggest that MDHB significantly extends the lifespan of *C. elegans* without causing a reduction in production, and MDHB regulates the lifespan of worms by both a *W06A7.4*-dependent and *W06A7.4*-independent mechanism. Besides, MDHB inhibits the expression of *daf-2*/IIS, while it upregulates the expression of the *daf-16* gene. Our genetic analysis shows that MDHB promotes *daf-16*/*FoxO* nuclear localization, thereby modulating the expression of *daf-2/daf-16* target genes that are positively associated with stress tolerance and lifespan regulation, including the upregulation of genes encoding the antioxidant enzymes. Putting all this together, we conclude that MDHB acts as a modulator of the *daf-2/daf-16* pathway to enhance resistance to oxidative stressors, ultimately prolonging the adult lifespan of *C. elegans*.

## 2. Results

### 2.1. Exposure to MDHB during Adulthood Extends the Mean Lifespan of Wild-Type C. elegans

**Figure 1:**
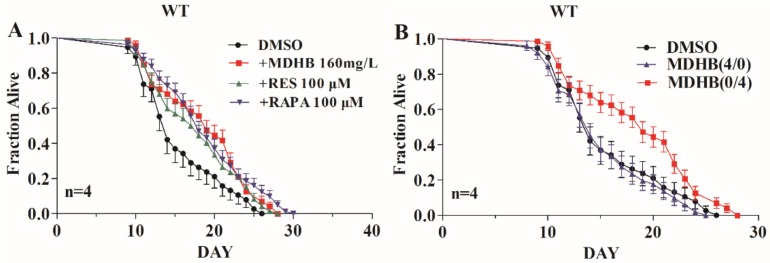
Methyl 3,4-dihydroxybenzoate (MDHB) extends adult life span of wild-type nematodes. (**A**) Survivorship curve of wild-type (WT) nematodes under different treatments. 100 μM resveratrol (+RES) and 100 μM rapamycin (+RAPA) were taken as the positive controls, separately. It was found that treatment with MDHB (Methyl 3,4-dihydroxybenzoate) (160 mg/L), resveratrol (100 μM) and rapamycin (100 μM) could significantly (*p* < 0.05) extend the mean lifespan of animals compared to the Normal control group. (**B**) Worms were exposed to MDHB (160 mg/L) from conception to L4 (+MDHB 4/0), or to MDHB from L4 to death (+MDHB 0/4). External drug concentrations are shown in milligrams per liter for MDHB. All data are described as mean ± S.E.M, the statistical details are summarized in Table 1.

In our previous study, worms cultured in an NGM (nematode growth media) medium containing 160 mg/L MDHB displayed the largest lifespan extension [13]. It was found that 160 mg/L MDHB extended the mean adult lifespan of worms from 15.47 ± 0.118 days to 18.41 ± 0.323 days (+19%, *p* < 0.01), more than the positive drug resveratrol group (+13.5%, *p* < 0.05) (Figure 1A and Table 1). A lifespan assay of worms pretreated with 160 mg/L MDHB from gestation to the fourth larval (L4) stage (MDHB 4/0) or from the L4 stage to death (MDHB 0/4) was conducted to investigate the action stage where MDHB worked to enhance lifespan. Exposure to MDHB from conception to fourth larval stage (MDHB 4/0) did not affect the lifespan of worms, while exposure to MDHB from L4 stage to death (MDHB 0/4) prolonged the adult lifespan to 18.41 ± 0.323 days (+19%, *p* < 0.01) (Figure 1B and Table 1). These findings show that MDHB functioned after the L4 stage to extend lifespan and had no effect on the developmental stage.

### 2.2. MDHB Reduces Lipofuscin Accumulation

**Figure 2:**
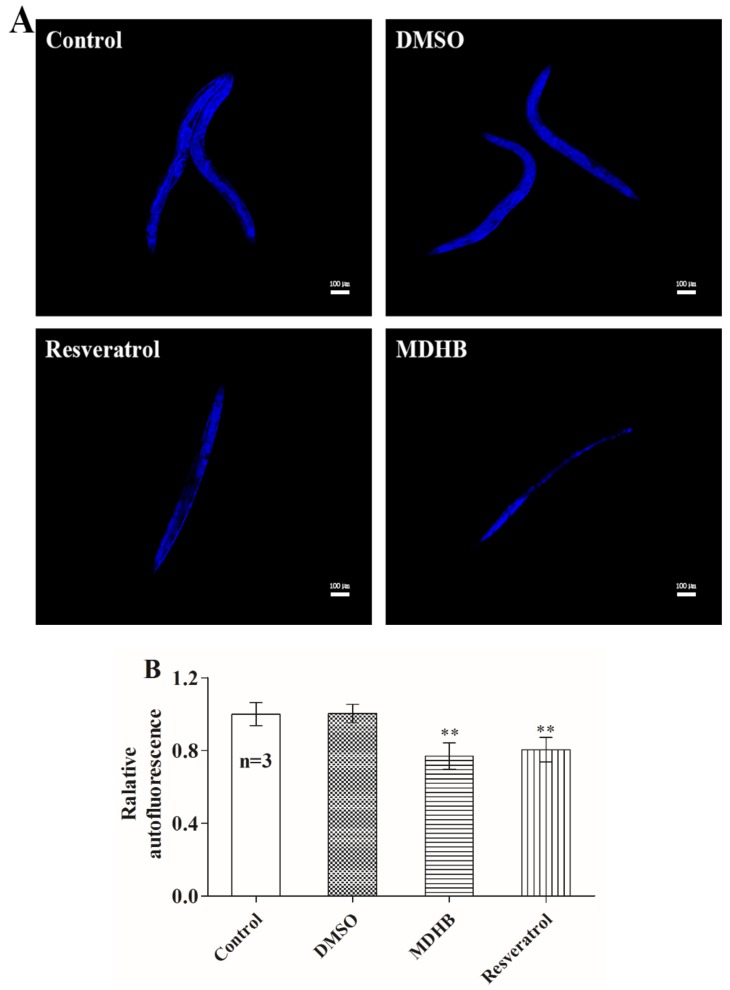
Effect of MDHB on lipofuscin accumulation of wild-type *C. elegans*. (**A**) Typical fluorescence images of worms in untreated control, Dimethyl sulfoxide (DMSO)-treated, resveratrol-treated and MDHB-treated worms are shown. (**B**) Effect of MDHB on intracellular lipofuscin accumulation in wild-type *C. elegans* at day 10. Fluorescence intensities of the lipofuscin fluorescence of 30 worms from different groups were analyzed by the ImageJ soft. Data are shown as means ± S.E.M comparing to the untreated control (OD-background/mm^2^ = 465.32 ± 31.56; set to 1). Statistical significance of differences between treated and control groups were described significant at ** *p* < 0.01 by one-way ANOVA with LSD post hoc test. ANOVA, analysis of variance. This experiment was performed three times.

Animals exposed to 160 mg/L MDHB had a reduced accumulation of lipofuscin, known as a biomarker for cellular damage and an autofluorescent protein that is cumulative in the progress of senescence [21,22]. Compared to untreated controls, intestinal lipofuscin levels at day 10 were reduced by 23.2% in MDHB-treated animals (*p* < 0.01) and 20% in resveratrol-treated groups (*p* < 0.01) (Figure 2A,B). Generally, lifespan extension of nematodes was related to decreased lipofuscin. Consequently, MDHB-induced reduction of lipofuscin demonstrated that MDHB could decrease oxidative protein damage, ultimately leading to a decelerated aging process in nematodes.

### 2.3. Effects of MDHB on SOD Activity in C. elegans

**Figure 3:**
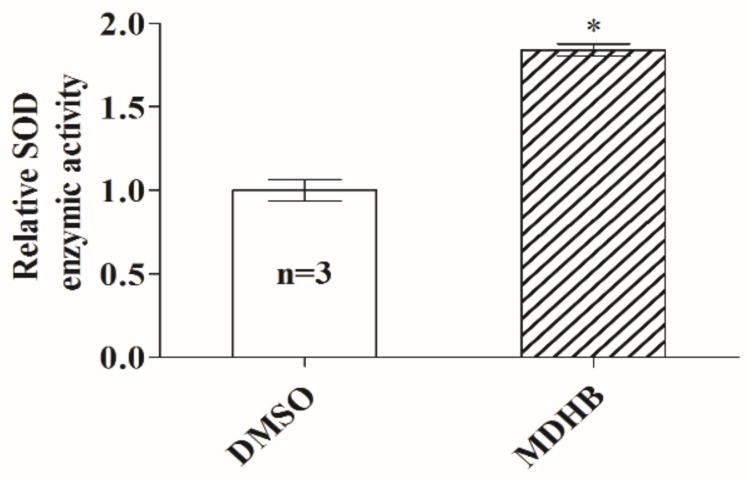
Effect of MDHB on superoxide dismutase (SOD) activity of wild-type *C. elegans*. Error bars represent the standard deviations (SD), and differences between the DMSO (control) and MDHB groups were described significant at * *p* < 0.05 by one-way ANOVA. This experiment was performed thrice.

MDHB is known to protect wild-type animals against thermal pressure [13]. Superoxide dismutase (SOD), an important antioxidant enzyme in organisms, plays a vital role in the elimination of some harmful substances produced in the metabolic process, like reactive oxygen species (ROS). In this study, we found that worms pretreated with 160 mg/L MDHB showed higher SOD activity compared to the DMSO (dimethyl sulfoxide) treated groups (Figure 3). That is to say, the antioxidant activity of MDHB could be a substantial contributor to its lifespan-extending activity in worms. Conducting further experiments on the effects of antioxidant MDHB in higher organisms would be indispensable in the late stage of this research.

### 2.4. MDHB Extends the Lifespan of W06A7.4 Loss-of-Function Mutants

**Figure 4:**
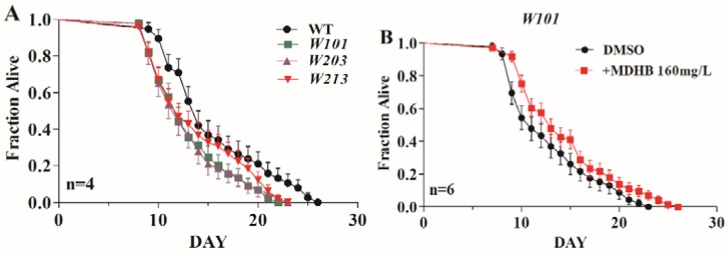

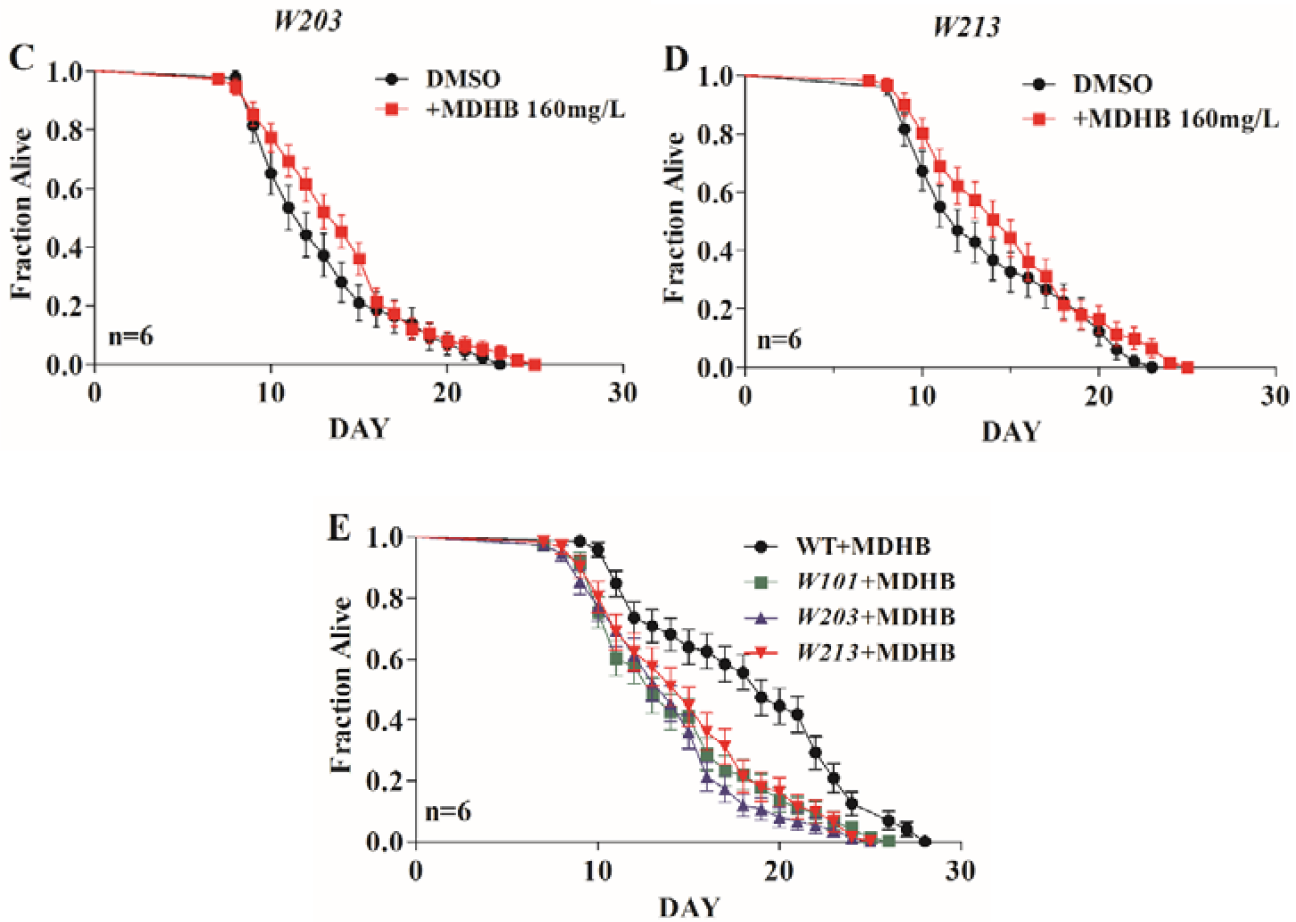
Effects of MDHB on the mean lifespan of *W06A7.4* loss-of-function mutations (*W101*, *W203*, and *W213* strains). (**A**) Survival of the three *W06A7.4* mutations (*W101*, *W203*, and *W213*) compared to wild-type animals. The mutated worms (*W101*, *W203*, and *W213*) had shorter lifespans than the wild-type N2 worms, *p* < 0.05. (**B**–**D**) Survival of mutated worms with *W06A7.4* gene knocked out, with and without MDHB treatment. Treatment with MDHB notably enhanced mean lifespan of three *W06A7.4* loss-of-function mutants, *W101* (+11.6%, *p* < 0.05), *W203* (+7.9%, *p* < 0.05), and *W213* (+9%, *p* < 0.05). (**E**) Survival of three *W06A7.4* loss-of-function mutations (*W101*, *W203*, and *W213* strains) and wild-type animals exposed to MDHB. Data are represented as mean ± S.E.M; statistical details are summarized in Table 1.

MDHB might extend the lifespan of wild-type *C. elegans* via the *W06A7.4* gene [13]. Therefore, three *W06A7.4* gene loss-of-function strains (*W101*, *W203*, and *W213*) were used to investigate the relationship between the *W06A7.4* gene and the pro-longevity action of MDHB in worms. It was found that wild-type worms (15.47 ± 0.118 days) lived longer than *W101* strains (13.30 ± 0.240 days, *p* < 0.05), *W203* strains (13.01 ± 0.260 days, *p* < 0.05), and *W213* strains (13.78 ± 0.270 days, *p* < 0.05) (Figure 4A and Table 1). However, our results show that exposure to MDHB extended the lifespan of all three *W06A7.4* loss-of-function mutants (*W101*, *W203*, and *W213* strains) (Figure 4B–D and Table 1), but the percentage change caused by MDHB was less than that in wild-type worms (Figure 4E and Table 1). Thus, we report that the longevity action of MDHB may partly require *W06A7.4*, and partial MDHB-induced longevity is independent of the *W06A7.4* gene in worms.

### 2.5. MDHB Does Not Appear to Lengthen Lifespan via Caloric Restriction

**Figure 5:**
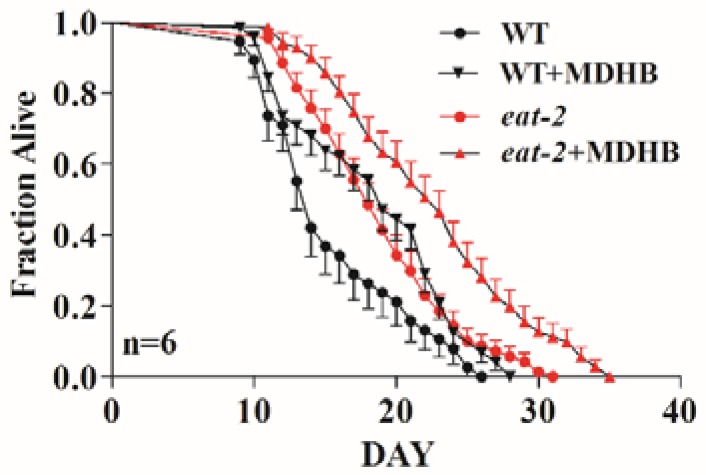
Effects of MDHB on the lifespan of *eat-2* (ad1113) mutants. MDHB notably prolonged lifespan of *eat-2* (ad1113) mutations from 18.77 ± 0.265 days to 22.60 ± 0.316 days (+20.4%, *p* = 0.039). Each experiment was repeated at least six times. The data are represented as mean ± S.E.M, the statistical details are generalized in Table 1.

Lifespan is modulated by various genetic and environmental elements; among them, caloric restriction is recognized as an irrefutable environmental modulator of lifespan in many species, including *C. elegans* [23,24,25,26]. Here, there was no significant difference in fat storage, food intake, and body patterning, because worms treated with MDHB did not become thin or starved. Furthermore, MDHB could extend the lifespan of wild-type worms without the reduced fecundity that usually occurs with nutrient limitation [13]. Combining the results, MDHB remarkably prolonged the mean lifespan of *eat-2* (ad1113) mutations, a genetic model of dietary restriction (DR), from 18.77 ± 0.265 days to 22.60 ± 0.316 days (+20.4%, *p* = 0.039) (Figure 5 and Table 1). Above all, we report that MDHB does not appear to enhance lifespan via caloric restriction.

### 2.6. MDHB Prolongs the Lifespan of C. elegans Partially through the daf-2/daf-16 Signaling Pathway

**Figure 6:**
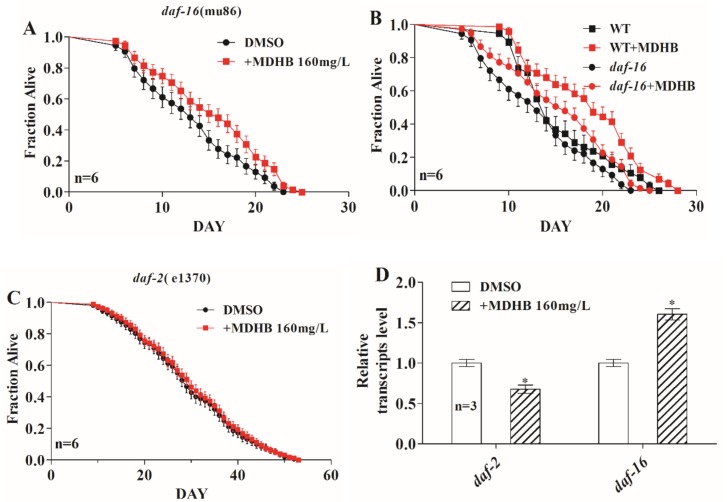
Effects of MDHB on the lifespan of *daf-2* and *daf-16* loss-of-function mutants. (**A**) Effects of MDHB on the lifespan of *daf-16* (mu86) mutations. (**B**) Effects of MDHB on the lifespan of Wild-Type (WT) animals and *daf-16* (mu86) mutants. (**C**) on the *daf-2* (e1370) mutants. (**D**) Quantification of MDHB exposure on the expression levels of *daf-2* and *daf-16* by RT-PCR (real-time polymerase Chain Reaction) in wild-type nematodes. Error bars represent the standard deviations (SD), and differences between the DMSO (control) and MDHB groups were described significant at * *p* < 0.05 by one-way ANOVA. *actin*-1 was used as an endogenous control, and the mRNA expression were determined by real-time PCR using the 2^−ΔΔ*C*t^ method, * *p* < 0.05.

The insulin/insulin-like growth factor (IIS) signaling pathway plays a vital role in lifespan regulation and other important biological processes in *C. elegans* and other creatures. The IIS pathway is modulated by insulin-like peptide ligands that bind to the insulin/IGF-1 transmembrane receptor (IGFR) ortholog *daf-2*. *daf-2*/IGFR regulates the activity of a conserved phosphoinositide 3-kinase (PI3K)/Akt kinase cascade, ultimately leading to the modulation of a forkhead family transcription factor, *daf-16*, that controls most of the functions of the IIS pathway [27,28,29]. Here, we found that MDHB enhanced the longevity of the *daf-16* loss-of-function mutations (+7%, *p* < 0.05) (Figure 6A and Table 1), but less than the percentage change in wild-type animals (+19%, *p* < 0.01) (Figure 6B and Table 1), indicating that part of the MDHB-induced lifespan extension is independent of *daf-16* and part of the action may demand *daf-16*. Moreover, there was no obvious difference in the mean lifespan between *daf-2* (e1370) mutations exposed to 160 mg/L MDHB or not (Figure 6C and Table 1). Furthermore, we found that worms with MDHB treatment showed a significantly lower expression of *daf-2*, but a higher expression of *daf-16* (Figure 6D). Taken together, these findings indicate that MDHB may prolong the lifespan of nematodes partially through the *daf-2*/*daf-16* signaling pathway.

### 2.7. MDHB Promotes daf-16/FoxO Nuclear Localization

**Figure 7:**
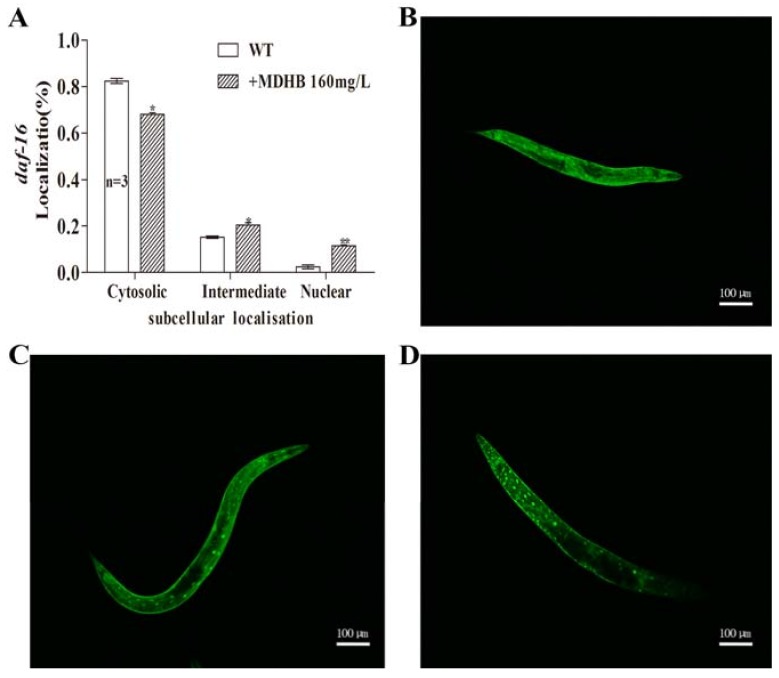
MDHB induced nuclear localization of *daf-16*. (**A**) Data from different group exposure to 160 mg/L MDHB or not are described as means ± S.E.M to show the percentile of animals displaying cytosolic, intermediate or nuclear of localization. Statistical significance among animals of the control groups and MDHB groups was analyzed by one-way ANOVA followed by Bonferroni (post-hoc) correction (* *p* < 0.05, ** *p* < 0.01). (**B**–**D**) Micrographs elucidate typical *daf-16* localization in TJ356 transgenic strains. (**B**) *daf-16* mainly locating in the cytoplasm, (**C**) mainly locating in cytoplasm and nucleus, (**D**) mainly in the nucleus. This experiment was repeated thrice.

Suppressing the *daf-2* signaling pathway transferred *daf-16* from cytoplasm into nucleus, where it modulates the expression of genes that are known to be associated with longevity regulation and diverse cellular stress responses [16]. We visualized the effect of MDHB on *daf-16* intracellular location through a specific transgenic strain (TJ356) that included the *daf-16*::GFP (green fluorescent protein) reporter construct integrated in the genome. Exposure to 160 mg/L MDHB induced 11.5% nuclear accumulation of *daf-16*::GFP in nuclei, but only 2.4% in worms in the untreated control groups (Figure 7), suggesting that MDHB might act upstream of *daf-16* and enhance its nuclear translocation. These results strongly indicate that *daf-16* is an indispensable part of the action of MDHB-induced promotion of antioxidant ability in nematodes, similar to other phenolic antioxidants.

### 2.8. Effects of MDHB on the Expression of daf-2/daf-16 Target Genes

**Figure 8:**
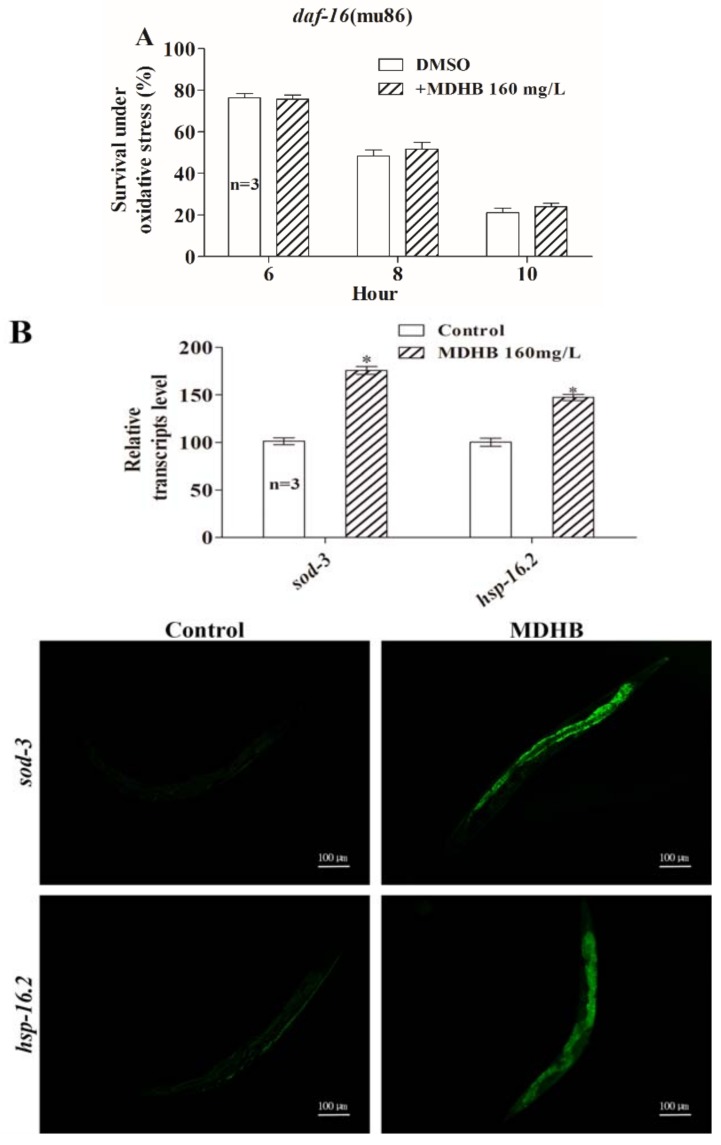

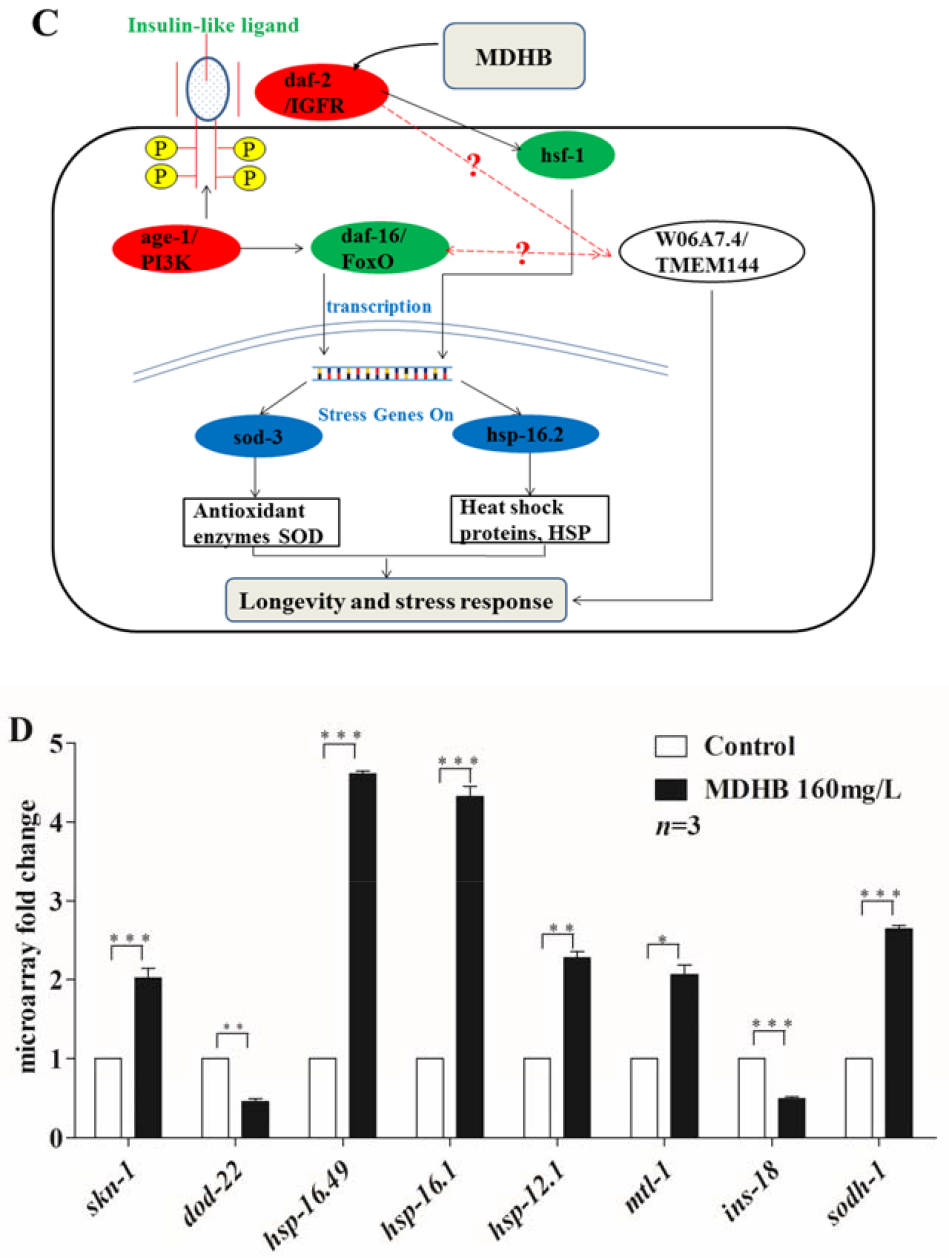
Influence of MDHB on the expression of *daf-2/daf-16* target genes. (**A**) Influence of MDHB on survival rates of *daf-16* (mu86) mutants under oxidative stress conditions induced by H_2_O_2_. MDHB did not enhance stress resistance in *daf-16* (mu86) mutants. (**B**) Influence of MDHB on the expression of *hsp-16.2* and *sod-3* genes under normal conditions. Green fluorescent protein (GFP) was tested and quantified using a fluorescence multi-reader, and analyzed by the standard Student *t*-test. (**C**) A drawing of *daf-2/daf-16* signaling and its mammalian homologues shows the supposed mechanism of action of MDHB in *C. elegans.* Active IIS promotes the phosphorylation-dependent cytoplasmic sequestration of the transcription factors *daf-16/FoxO* and *hsf-1*. The insulin/IGF-1 receptor ortholog *daf-2* and other pathway components that promote IIS are colored red, and molecules that either antagonize IIS or are antagonized by IIS are colored green. Genes that are promoted by *daf-16/FoxO* are colored blue. Molecular, which may either promote or antagonize the activity of its target genes, are marked with black arrows. Genes that either antagonize *daf-2/daf-16* or are antagonized by *daf-2/daf-16* are marked with red arrows. Abbreviations: IIS, insulin/insulin-like growth factor-1 signaling; IGFR, Insulin-like growth factor 1 receptor; PI3K, phosphoinositide 3-kinase; *FoxO*, forxhead box protein O; P, the phosphate groups. (**D**) Influence of MDHB on the expression of partial *daf-2*/*daf-16* pathway targets. The effects of MDHB on the expression of genes were detected through a microarray profiling analysis. * *p* < 0.05; ** *p* < 0.01; *** *p* < 0.001; ns: not significant.

In *C. elegans*, many genes and pathways are associated with oxidative stress response and longevity regulation (e.g., IIS signaling pathway, CR pathway). Previous research reported that an increase in oxidative stress tolerance was associated with prolonged lifespan in nematodes [19,30,31]. The result that MDHB protected wild-type worms from oxidative damage while it did not enhance stress resistance in *daf-16* (mu86) mutants (Figure 8A) suggested that *daf-16* is essential for the observed MDHB-mediated lifespan enhancement under oxidative stress resistance. Strains CF-1553 and CL-2070 were used to examine the transcriptional levels of *sod-3* and *hsp-16.2* that are positively related to lifespan and oxidative stress [31,32]. Here, the expression of GFP fused *hsp-16.2* or *sod-3* was significantly up to 47 ± 5.75% and 76 ± 7.05% with MDHB treatment (Figure 8B, *p* < 0.001). We report that MDHB promotes *daf-16* entering into the nucleus and enhances the transcriptional levels of *sod-3* and *hsp-16.2* to protect worms from oxidative stress. Above all, these results put forward a supposed mechanism of action of MDHB in *C. elegans* (Figure 8C).

To further investigate whether the improved oxidative stress resistance of *C. elegans* pretreated with MDHB depends on *daf-16*–regulated activity, a microarray profiling analysis was used to examine the effects of MDHB on the following *daf-2/daf-16* targeted genes: *skn-1* [33,34], *cst-1* [35], *dod-22* [36], *hsp-12.1*, *hsp-16.1*, *hsp-16.49* [36], *mtl-1* [36], *ins-18* [36], and *sodh-1* [37]. The profiles of the differentially expressed genes (MDHB treatment vs. DMSO treatment, fold change >2 or <0.5) were obtained by analyzing the MDHB-regulated genes on the 6th day respectively within the three batches of the samples. The details about these genes are summarized in Table 2. 8 genes were confirmed by matching differentially expressed genes on the 6th day (Figure 8D). The corresponding functional annotations of these genes are shown in Table 2. Expression of *skn-1*, *hsp-12.1*, *hsp-16.1*, *hsp-16.49*, *mtl-1* and *sodh-1* was significantly increased (fold change >2, *p* < 0.05), while *dod-22* and *mtl-2 significantly* decreased (fold change >2 or <0.5, *p* < 0.05) (Figure 8D and Table 2). Together, these results suggest that MDHB prolongs the lifespan of *C. elegans* at least partially through the *daf-2*/*daf-16* pathway, which is connected to oxidative stress resistance and longevity regulation.

## 3. Discussion

We previously reported that MDHB has antioxidant, neurotrophic, and neuroprotective effects [9,10,11,12]. Besides, MDHB extends the lifespan of *C. elegans* [13]. However, the mechanism by which MDHB extends lifespan remains hard to determine. The studies described here were aimed at exploring the exact mechanisms of lifespan-extending activity of MDHB in *C. elegans*.

First, our results demonstrate that MDHB prolongs *C. elegans* lifespan and delays the age-related declines of physiological processes. Moreover, we observe that worms exposed to MDHB show higher SOD activity and reduced accumulation of intracellular lipofuscin, which are associated with longevity regulation in worms. These findings raise a crucial question: what is the exact mechanism of lifespan-extending action of MDHB in *C. elegans*? 

As previously reported, a variety of elements have been demonstrated to prolong the lifespan of worms, and the studies described here have clarified the relationship between MDHB and these elements.

Among the numerous factors that regulate lifespan, caloric restriction (CR) is recognized as an indisputable environmental modulator of lifespan in many species, including *C. elegans* [23,24]. CR prolongs lifespan and can be caused by a mutation of the *eat-2* gene, which plays a vital role in pharyngeal pumping [25,26]. MDHB obviously prolonged the lifespan of *eat-2* (ad1113) mutations (20.4%) (Figure 5 and Table 1), suggesting that the primary mechanism of MDHB-induced longevity in worms is not caloric restriction. Besides, wild-type worms pretreated with MDHB were not nutrient-limited, as the animals did not display obvious differences in reproduction, fat storage, or somatotype (showing thin or starved) that always occur with CR [26].

As previously reported, the mechanism of MDHB in lifespan extension might be associated with the *W06A7.4* gene in worms. Here, we report that three *W06A7.4* loss-of-function mutations (*W101*, *W203*, *W213*) had shorter lifespans than wild-type N2 worms. However, the lifespan of three *W06A7.4* mutations could be further extended by MDHB, but the percentage change caused by MDHB in *W06A7.4* mutations was lower than that in wild-type nematodes. One explanation is that the reduced effect of MDHB is in agreement with other probabilities, including the detrimental consequences of pleiotropy caused by combining a mutation with MDHB. In addition, these results indicate that part of the longevity action of MDHB may require *W06A7.4*, and part of MDHB-induced lifespan-extending activity is independent of the *W06A7.4* gene.

The insulin/IGF signaling pathway is a classical pathway that governs compressive capacity, dauer formation, and longevity [14,27]. Loss-of-function *daf-16* mutations reduce lifespan and suppress lifespan extensions caused by mutations in upstream signaling pathway genes such as *daf-2*. Worms with MDHB treatment showed a significantly lower expression of *daf-2*, but a higher expression of *daf-16* (Figure 6D). We also observed that MDHB exposure significantly prolonged the lifespan of *daf-16* (mu86) mutants (7%), though less than the percentage change in wild-type animals (19%), indicating that MDHB regulates the lifespan of worms by both *daf-16*–dependent and *daf-16*–independent mechanisms. However, treatment with MDHB did not further extend the lifespan of *daf-2* (e1370) mutants with defects in *daf-2*, revealing that *daf-2* is not related to the mechanism of MDHB-induced long life in *C. elegans*.

We previously found that part of the lifespan-extending activity of MDHB in worms may need *W06A7.4* and *daf-16*. Combining the result that *daf-2* is not connected to the longevity action of MDHB in worms, these results suggest that MDHB at least partly works as a *daf-2*/*daf-16* modulator to extend lifespan in *C. elegans*. Furthermore, this paper puts forth the idea that the *W06A7.4* gene may function downstream of *daf-16* or in parallel to *daf-16*/*FoxO*, but in the *daf-2*–mediated IIS pathway to regulate the adult lifespan of *C. elegans*. That is to say, the relationship between *W06A7.4* and *daf-2/daf-16* remains to be seen in further experiments (Figure 8C).

Aging has been related to oxidative stress resistance, and various studies have proved that lifespan modulation affects resistance to oxidative stress [19]. Accordingly, we performed an oxidative stress resistance assay to analyze the effects of MDHB on oxidative stress, and our studies showed that MDHB protected wild-type animals against oxidative damage, while it did not extend mean lifespan of *daf-16* (mu86) loss-of-function mutations (*p* > 0.05) under oxidative stress. This finding indicates that *daf-16* leads to the protective effect of MDHB in worms. Moreover, worms pretreated with MDHB showed enhanced *daf-16* nuclear translocation and promoted expression of GFP guided by *hsp-16.2* or *sod-3*, which functions downstream of *daf-16*. *sod-3* is a typical scavenger enzyme of ROS in oxidative stress, and it catalyzes the disproportionation of active superoxide anions to molecular oxygen. One explanation is that the actions of MDHB are modulated, at least partially, by activating *hsp-16.2* and *sod-3* genes that are positively related to lifespan regulation and stress resistance. We suggest that a readjustment of MDHB on *daf-16* leads to the verified antioxidant effects of MDHB in *C. elegans*.

To further investigate whether the improved resistance to oxidative stress of *C. elegans* pretreated with MDHB depends on *daf-2/daf-16*–regulated activity, a microarray profiling analysis was conducted to examine the effects of MDHB on the following *daf-2*/*daf-16* target genes that are involved in oxidative stress response and ageing [34]. It was found that the expression of *skn-1*, *hsp-12.1*, *hsp-16.1*, *hsp-16.49*, *mtl-1*, and *sodh-1* was significantly increased, while *dod-22* significantly decreased in worms exposed to MDHB, indicating that *daf-16* is necessary for the antioxidant effect of MDHB in *C. elegans*. Earlier research indicated that *daf-16* functioned with other genes or transcription factors to regulate stress resistance and longevity in *C. elegans* [36]. In *C. elegans*, *skn-1* is homologous to mammalian nuclear factor erythroid-related factor (Nrf) transcription factors. *skn-1* functions in parallel to *daf-16* to regulate stress resistance and aging in worms [33,34]. *ins-18*, a *daf-2* antagonist, functions downstream of *daf-16* to regulate the lifespan of *C. elegans* [36]. Lifespan extension was also found in *ins-18* loss-of-function mutations [36]. Under conditions of stress or reduced *daf-2* signaling, *daf-16* entered into the nucleus, then modulated the transcription levels of genes associated with oxidative stress response and aging. Furthermore, MDHB exposure upregulated the expression of genes encoding antioxidant enzymes such as superoxide dismutase (*sod-3*) [36] metallothionein (*mtl-1*) [36], and sorbitol dehydrogenase (*sodh-1*) [37], which were predicted to have oxidoreductase activity [36]. Loss of function *dod-22* increases the lifespan of worms [36]. MDHB downregulated the expression of *dod-22* in worms. In addition, *daf-16* functions with *hsf-1* to regulate the expression of genes encoding the small heat-shock proteins. Upregulation of *hsp-16.1*, *hsp-16.49*, and *hsp-12.1* were found in worms with MDHB treatment.

Taken together, these results suggest that MDHB enhances resistance to oxidative stressors and increases lifespan in nematodes at least partially by modulating *daf-2*/*daf-16*. However, more experiments must be conducted to investigate the underlying mechanisms of MDHB induced lifespan-extending action as the molecular targets of MDHB are not yet fully understood.

## 4. Materials and Methods

### 4.1. Chemicals

MDHB (Tokyo Chemical Industry, Tokyo, Japan) was dissolved in DMSO (Sigma-Aldrich, St. Louis, MO, USA) at a concentration of 160 mg/mL and conserved it at 4 °C. The purity of MDHB was confirmed to be more than 98%.

### 4.2. General Methods and Strains

N2 (wild-type), *eat-2* (ad1113), *daf-16* (mu86), *daf-2* (e1370), TJ356 (zIs356 IV), CF1553 (muIs84), CL-2070 (dvIs70) strains were used in this study. All nematodes were cultivated at 20 °C on culture dishes (60 mm) containing nematode growth medium and a thick lawn of *Escherichia coli* OP50 bacteria [5]. N2 strain and streptomycin-resistant *E. coli* OP50 were amicably provided by Xiao-Chen Wang (National Institute of Biological Sciences, Beijing, China). Other strains were kindly provided by from Pei Zhong (School of Pharmaceutical Science, Sun Yat-sen University, Guangzhou, China). Coeval nematodes gained by treatment with alkaline hypochlorite [38].

### 4.3. W06A7.4 Gene Knockout Through a CRISPR-Cas9 System

Earlier experiments confirmed that the *W06A7.4* gene might be associated with lifespan-extending activity of MDHB in *C. elegans*. Three *W06A7.4* gene loss-of-function mutations were gained from the wild-type worms by a CRISPR-Cas9 gene editing system, including two mutant strains (*W203*, *W213*), in which two transcripts, *W06A7.4*b and *W06A7.4*a, were disrupted, and another mutant (*W101*) had only a transcript *W06A7.4*a mutation [5,38,39,40,41,42,43].

### 4.4. Lifespan Assay

Animals were cultivated at 20 °C on culture dishes (60 mm) containing nematode growth medium with drugs or not and a thick lawn of *Escherichia coli* OP50. To obtain coeval nematodes, 10~20 pregnant nematodes were picked up to a fresh culture dish in the absence or presence of 160 mg/L MDHB for egg-laying about 4–6 h, or treated with alkaline hypochlorite [44]. Synchronous L4 larvae were defined as day 0 and observed every day until death. Animals were transferred to fresh plates every day till the end of propagative period, and then transferred to new dishes about every 4 days thereafter. Animals displaying no autonomic movement or response when touched were mounted as dead. Censored data containing dead worms that procreated progeny internally, and manifested an extruded gonad or dehydration owing to wall climbing [3].

### 4.5. Lipofuscin Accumulation Assay

Age-synchronous L4 larvae were cultured in the presence of 5-Fluorouracil (100 µM) and maintained with or without 160 mg/L MDHB for 10 days for intracellular lipofuscin accumulation test. Ten day old wild-type nematodes were pipetted on a microscope slide with 2% agar pad and anesthetized with levamisole (5 mM, 3 min). An Olympus X71 fluorescent microscope (Olympus X71, Olympus, Tokyo, Japan) was applied to capture the autofluorescence of lipofuscin [45]. The experiment has been repeated thrice. The standard Student’s *t*-test was employed for statistical analysis.

### 4.6. Measurement of SOD Activity

To assess superoxide dismutase (SOD) activity, wild-type worms were cultured in NGM plates containing 160 mg/L MDHB or not for 6 days. Animals on the sixth day of adulthood were collected from different plates with M9 buffer and washed thrice. Afterward, the harvested animals were suspended in M9 buffer and homogenized on ice [46]. The EnSpire Multimode Plate Reader (PerkinElmer, Waltham, MA, USA) was used to evaluate SOD activity according to an SOD assay kit (WST-1 method) (Nanjing Jiancheng, Nanjing, China).

### 4.7. Oxidative Stress Resistance Assay

Oxidative stress resistance of worms was analyzed according to the method described formerly with a bit of modification. Briefly, hydrogen peroxide (H_2_O_2_) induced oxidative stress assays were performed at 20 °C as for lifespan assays, except hydrogen peroxide was added to NGM medium at final concentration of 3 mM. Synchronous L4 larvae were cultured under different conditions (control and 160 mg/L MDHB groups) for 72 h at 20 °C. The 3-day Adult hermaphrodites were incubated at 20 °C on NGM plates containing 3 mM H_2_O_2_, an intracellular free-radical-generating compound, and a thick lawn of *Escherichia coli* OP50 to prevent nematodes from starvation response. Afterwards the survival ship of worms were recorded every hour until all nematodes died [47]. The dead worms were counted as described in the method of lifespan assay. Briefly, survivals of worms was monitored by touch-provoked movement method [48]. The experiment was repeated thrice, independently.

### 4.8. Expression of sod-3 and hsp-16.2

Age-synchronized CF1553 worms steadily expressing a *sod-3*::GFP fusion protein and CL2070 worms steadily expressing a *hsp-16.2*::GFP fusion protein were treated with 160 mg/L MDHB under normal condition at 20 °C for 6 d. Worms were placed on the microscope slide coated with 2% agar anaesthetised with levamisole (5 mM, 3 min). GFP were monitored with a confocal microscope (Olympus X71, Olympus, Tokyo, Japan). For quantitative analysis of GFP expression, 20 worms were randomly selected from both experimental groups and placed into a 96-well plate (a single worm in each well). The GFP fluorescence intensity of each worm from different groups was monitored with a confocal microscope (Zeiss, Göttingen, Germany) and quantified with a fluorescence multi-reader (PerkinElmer, USA; *n* = 20) [49]. This experiment was repeated three times. The standard Student’s *t*-test was employed for statistical analysis.

### 4.9. daf-16 Localization

Age-synchronized strain TJ356 worms were treated with 160 mg/L MDHB at 20 °C for 6 days. Then, day-6 TJ356 nematodes were mounted on the glass slides coated with 2% agar anaesthetised with levamisole (5 mM, 3 min), and *daf-16* location of worms were determined through a fluorescence microscope (Zeiss, Göttingen, Germany) at 100× magnification. *daf-16* localization of each worm were classified into 3 types (cytosolic, intermediate and nuclear) in consideration of the main location of *daf-16*::GFP [49]. Intracellular location of the *daf-16*::GFP of each worm was analysed using the ImageJ software (NIH, Bethesda, MA, USA).

### 4.10. Microarray Profiling Analysis

Age-synchronized wild-type L4 larvae were cultured under different conditions (untreated control, 1% DMSO, and 160 mg/L MDHB groups) in the presence of 5-fluorouracil (100 µM) at 20 °C for 6 days. Then, day-6 adult worms of different groups were collected for microarray profiling analysis (Shanghai Biotechnology Corporation, Shanghai, China). Total RNA was extracted using TRIZOL reagent (Cat#15596-018, Life Technologies, Carlsbad, CA, USA) following the manufacturer’s instructions and checked for a RIN (RNA INtegrity) number to inspect RNA integrity by an Agilent Bioanalyzer 2100 (Agilent Technologies, Santa Clara, CA, USA). Qualified total RNA was further purified by RNeasy Micro Kit (Cat #74004, Qiagen GmBH, Hilden, Germany) and RNase-Free DNase Set (Cat #79254, Qiagen) and then used for microarray analysis.

## 5. Conclusions

Here, we show that a readjustment of MDHB on *daf-2/daf-16* leads to verified lifespan-extending action of MDHB in *C. elegans*. MDHB inhibits the expression of *daf-2*/IIS, while it upregulates the expression of the *daf-16* gene. Then, MDHB promotes *daf-16*/*FoxO* nuclear localization, thereby modulating the expression levels of its target genes that are positively associated with stress tolerance and lifespan regulation, including the upregulation of genes encoding the antioxidant enzymes *hsp-16.2*, *hsp-16.49*, *hsp-16.41*, *mtl-1*, and *sod-3*. Given the evidence, we conclude that MDHB acts as a modulator of the *daf-2*/*daf-16* pathway to enhance resistance to oxidative stressors, ultimately prolonging the adult lifespan of *C. elegans*.

## Figures and Tables

**Table 1:** Summary of mean life-spans of *C. elegans*.

Genotype	Treatment	Mean ± SEM (Days)	% Change	N (Censored)	EXP	*p*	95% CI for Mean	
							*Lower Bound*	*Upper Bound*
WT, 20 °C	None	15.47 ± 0.118		1132 (168)	13		15.11	15.83
	MDHB (0/4) 160 mg/L	18.41 ± 0.323	+19	346 (54)	4	0.008	17.76	19.06
	MDHB (4/0) 160 mg/L	15.02 ± 0.235	−2.9	372 (28)	4	0.618	14.56	15.49
	RES 100 μM	17.56 ± 0.316	+13.5	352 (48)	4	0.012	16.94	18.18
	RAPA 100 μM	18.76 ± 0.332	+21.3	367 (33)	4	0.003	18.11	19.40
*W101*	None	13.30 ± 0.240	−16	512 (88)	6		12.83	13.78
	MDHB	14.84 ± 0.276	+11.6	489 (111)	6	0.018	14.30	15.38
*W203*	None	13.01 ± 0.260	−16	508 (92)	6		12.50	13.52
	MDHB	14.03 ± 0.235	+7.9	492 (108)	6	0.042	13.57	14.52
*W213*	None	13.78 ± 0.270	−11	522 (78)	6		13.25	14.32
	MDHB	15.01 ± 0.281	+9	501 (99)	6	0.038	14.46	15.56
*daf-16* (mu86)	None	13.15 ± 0.337	−15	546 (54)	6		12.55	13.75
	MDHB	14.07 ± 0.306	+7	523 (77)	6	0.036	13.41	14.72
*daf-2* (e1370)	None	29.24 ± 0.469	+89	532 (68)	6		28.32	30.16
	MDHB	30.06 ± 0.459	+2.8	513 (87)	6	0.512	29.16	30.96
*eat-2* (ad1113)	None	18.77 ± 0.265	+21.3	576 (24)	6		18.25	19.29
	MDHB	22.60 ± 0.316	+20.4	543 (57)	6	0.039	21.98	23.22

All strains were fed live *E. coli* OP50 and cultured at 20 °C. The concentrations were represented in milligrams per liter for methyl 3,4-dihydroxybenzoate (MDHB). Genotypes with no drug treatment are compared with line 1. Otherwise, comparisons are to the same genotype with no drug treatment. For these comparisons in the columns showing life-spans that were shown as Mean ± S.E.M (days) in column 3. Column 2, worms with or without drug treatment. N, the total number of worms were tested. EXP, the number of independent experiments.

**Table 2:** Effects of MDHB on the expression of partial *daf-2*/*daf-16*-dependent targets.

Cosmid No.	Gene Symbol	Brief Description	MDHB Treatment	
			Fold Change	*p* Value
T19E7.2	*skn-1*	orthologous to the mammalian Nrf (Nuclear factor-erythroid-related factor) transcription factors	2.01	<0.001
F55G11.5	*dod-22*	DUF141 domain of unknown function, high similarity to uncharacterized *C. elegans* K10D11.2	−2.22	0.0023
T22A3.2	*hsp-12.1*	a member of the small heat shock family of proteins	2.27	0.0045
T27E4.8	*hsp-16.1*	Member of the *C. elegans hsp-16* family	4.32	<0.001
T27E4.3	*hsp-16.49*	Hsp20/alpha crystallin family, similar to alpha-B crystalline	4.61	<0.001
K11G9.6	*mtl-1*	Metallothionein-related cadmium-binding protein	2.06	0.0083
T28B8.2	*ins-18*	Insulin-like protein of the type-β subfamily; may be a ligand for the *daf-2* receptor	−2.04	0.0421
K12G11.3	*sodh-1*	Alcohol dehydrogenase 1, have oxidoreductase activity	2.64	<0.001

## Abbreviations

MDHB	Methyl 3,4-dihydroxybenzoate
*C. elegans*	*Caenorhabditis elegans*
IIS	Insulin/Insulin-like growth factor-1 signaling pathway
*daf-16*	Orthology of the *FoxO* in *C. elegans*
*FoxO*	Forxhead box protein O
CR	Calorie restriction
DMSO	Dimethyl sulfoxide
ROS	Reactive oxygen species
SOD	Superoxide Dismutase
RES	Resveratrol
RAPA	Rapamycin
5-FuDR	5-fluorouracil deoxyribotside
*E. coli*	*Escherichia coli*
NGM	Nematode growth medium
LB	Luria-Bertani
RIN	RNA integrity
